# Physical Activity, BMI and Body Weight Perception Among 15-year-old Boys and Girls in Poland in The Light of International Comparisons

**DOI:** 10.34763/devperiodmed.20172103.235247

**Published:** 2017-10-28

**Authors:** Dorota Kleszczewska, Anna Dzielska, Hanna Nałęcz, Joanna Mazur

**Affiliations:** 1 Foundation of the Institute of Mother and Child, Warsaw, Poland; 2 Department of Child and Adolescent Health, Institute of Mother and Child, Warsaw, Poland

**Keywords:** adolescents, physical activity, BMI, body image, młodzież, BMI, aktywność fizyczna, obraz własnego ciała

## Abstract

**Background:**

The wrong subjective assessment of body mass may have consequences for the health, quality of life and proper functioning of adolescents in society.

**Aim:**

The purpose of the study is to provide a detailed analysis of the discrepancies between the objective and subjective assessment of body mass by 15-year-old boys and girls in Poland against the background of international statistics and to assess the effect of physical activity on the level and directions of these discrepancies.

**Material and methods:**

The data concerned 72037 schoolchildren from 42 countries, including 1484 in Poland. The measure of objective body mass was the BMI categorized according to Cole’s criteria. Research focused on the relation between the mean BMI and the subjective perception of their body mass by adolescents and the discrepancy in this assessment. In addition, the level of physical activity (MVPA) was taken into account.

**Results:**

In Poland the percentage of adolescents with overweight was lower than the international average and amounted to 12.3% vs. 15.2%. The percentage of 15-year-old boys with overweight in Poland is nearly three times higher than girls of the same age (18.7% vs. 6.5%) and is at the mean level for Health Behaviour in School-aged Children (HBSC) countries. According to international statistics, the percentage of adolescents who consider themselves overweight is 32.7%, which is two times higher than the percentage of actual excess body weight. Polish girls are systematically first in the ranking of those who consider themselves too fat. It was demonstrated that Polish girls with a normal BMI for their age who consider themselves too fat are on average slimmer than their peers in other countries. There is a correlation between Moderate-to-Vigorous Physical Activity (MVPA) and the accuracy of self-assessment of body mass and the type of mistakes made in that assessment, which is more clearly marked among boys.

**Conclusion:**

There are clear geographical and cultural differences in the level of discrepancies between the objective and subjective assessment of body mass among teenagers. Physical activity modifies these discrepancies.

## Streszczenie

**Wstęp:**

Nieprawidłowa ocena masy ciała może mieć wpływ na zdrowie, jakość życia i prawidłowe funkcjonowanie nastolatków w społeczeństwie.

**Cel pracy:**

Celem pracy jest pogłębiona analiza rozbieżności między obiektywną i subiektywną oceną masy ciała 15-letnich dziewcząt i chłopców w Polsce, na tle statystyk międzynarodowych oraz ocena wpływu aktywności fizycznej na poziom i kierunki tych rozbieżności.

**Materiał i metody:**

Badaniem w 42 krajach objęto 72 037 uczniów, w tym 1484 w Polsce. Obiektywną ocenę masy ciała określano wskaźnikiem BMI klasyfikowanym według kryteriów Cole’a. Badano związek pomiędzy średnim wskaźnikiem BMI, a subiektywną oceną masy ciała nastolatków oraz poziomem rozbieżności w tej ocenie. Ponadto wzięto pod uwagę poziom aktywności fizycznej (MVPA) badanych nastolatków.

**Wyniki:**

W Polsce odsetek nastolatków z nadwagą jest niższy od średniej międzynarodowej i wynosi 12,3% w porównaniu z 15,2%. Odsetek 15-letnich chłopców z nadwagą w Polsce jest prawie trzykrotnie wyższy niż odsetek dziewcząt w tym samym wieku (18,7% vs 6,5%) i jest na średnim poziomie dla państw HBSC. Według statystyk międzynarodowych odsetek młodych osób, które uważają się za zbyt grube, wynosi 32,7%, czyli dwa razy więcej niż odsetek młodzieży z rzeczywistą nadwagą. Polskie dziewczęta systematycznie zajmują pierwsze miejsce w rankingu dziewcząt, które uważają się za zbyt grube. Wykazano, że polskie dziewczęta z BMI w normie dla wieku, które uważają się za zbyt grube, są średnio szczuplejsze niż ich rówieśniczki z innych krajów. Potwierdzono związek pomiędzy aktywnością fizyczną (MVPA) a trafnością samooceny masy ciała oraz rodzajem popełnianych błędów. Związek ten jest wyraźniejszy wśród badanych chłopców.

**Wnioski:**

Istnieją wyraźne różnice geograficzne i kulturowe w poziomie rozbieżności pomiędzy obiektywną i subiektywną oceną masy ciała nastolatków. Aktywność fizyczna modyfikuje te rozbieżności.

## Introduction

The body mass index is one of the most important indicators of human health. The first measurements of weight and length are made a few minutes after birth and for many years it is one of the most frequently examined features used for assessing the development of a child’s, teenager’s [1] and ultimately adult’s [2] health. For many years not only the objective body mass but also its subjective assessment has been frequently undertaken in Polish, as well as international studies [3]. Self-assessment of body weight is a multi-dimensional construct considered in its cognitive, affective and behavioural aspects [4]. Inadequate body weight assessment may indicate dissatisfaction with one’s appearance resulting from the discrepancy between the perception of one’s figure and the perceived ideal. Subjective body mass assessment is particularly important in adolescence, because at this age it affects one’s overall satisfaction with life [5] and the proper social interaction of teenagers [6]. Dissatisfaction with one’s body is associated with low self-esteem, occurrence of mental health problems and disorders (depression, nutritional disorders) and somatic complaints. Excessive preoccupation with one’s appearance and body weight lead to behaviours aimed at changing one’s appearance that are harmful for health, such as elimination or unconventional diets, induced vomiting or use of laxatives. The results of many studies indicate that gender determines objective body weight assessment. Boys tend to overestimate their body weight more frequently. They want to be seen as bigger and more muscular. Conversely, girls tend to underestimate their body weight during assessment [7]. Dissatisfaction with one’s appearance increases with age among both genders but lasts longer among girls and young women.

For many years the results of successive rounds of international HBSC (Health Behaviour in School-aged Children) studies point out Poland as the country where girls have the least favourable assessment of their body weight [8]. Detailed national [9] and international [10] studies have shown that the high percentage of girls dissatisfied with their appearance increases with age, which [11] is not confirmed by objective measurements of the prevalence of overweight and obesity. In fact excess body weight actually affects to a greater extent boys and younger children [12]. Analyses are usually performed by classifying BMI (Body Mass Index) groups, i.e. underweight, normal weight, overweight, obesity [13]. Less attention is paid to BMI variability on a continuous scale, including variability within a group of adolescents without excess weight, taking into account the differences between countries.

The level of physical activity may be a factor which modifies the perception of one’s figure; on the one hand it influences one’s self-esteem [14] and on the other the distribution of muscle and fat tissue [15, 16] affecting the image of one’s figure and level of satisfaction with one’s appearance [17].

## Aim of the study

The purpose of the study is to provide a detailed analysis of the discrepancies between objective and subjective assessment of body weight among 15-year-old boys and girls in Poland in the light of international statistics the paper aims to assess the impact of physical activity on the level and directions of these discrepancies.

## Research questions

Five research questions were formulated:

- What is the distribution of BMI values as an objective measure of body weight among Polish 15-year-olds in the light of international data?
- What is the position of 15-year-old Polish boys and girls’ in international rankings in terms of subjective body weight self-perception?
- How often do discrepancies between objective facts and body weight self-perception occur among Polish adolescents on the basis of international comparisons?
- Does the BMI indicator demonstrate geographic differences (between countries) within groups of young people homogeneous in terms of self-perception of body weight?
- Does physical activity modify the level of discrepancy between the objective and self-perceived body weight among boys and girls?

## Material and methods

The analyses were performed on the basis of data collected during the last round of HBSC studies during the 2013/14 school year within the statutory activity of the Institute of Mother and Child. The data obtained from the international HBSC database in Bergen relate to 72 037 schoolchildren aged 14.5-16.5 years from 42 countries of Europe and North America. The average age of the respondents was 15.53±0.36 years. The size of the samples in individual countries varied from 320 in Greenland to 4973 in Canada. On average 1715±720 schoolchildren from this age group were covered by the poll in each country, including 1484 in Poland.

After taking into account the missing data for the key variables concerning objective assessment and subjective evaluation of the body weight of 61061 schoolchildren, they were enrolled for further analysis. In comparison with the original sample, 14.2% missing values were found concerning height and body weight (making it impossible to calculate BMI) and 2.1% concerning self-perception of body weight. The percentage of missing data concerning the BMI indicator in Poland was 6.0%, while in some other countries it reached 60% (Ireland, Scotland).

## Variables and indicators

### Excessive body mass index

The BMI was calculated on the basis of data concerning self-reported body weight and height obtained directly from the adolescents examined. The criteria of the International Obesity Task Force (IOTF) (so called Cole’s criteria) [18] were applied to identify overweight and obese young people. Cole’s indicator is the quotient between the current and standard body mass index: LMS = (BMI curr./BMI stand.) × 100 [%] [19]. For boys aged 15.5 years the cut-off point for overweight is BMI=23.6 kg/m, for obesity BMI=28.6 kg/sq.m.; for girls 24.17 kg/sq.m. and 29.29 kg/sq.m. [20, 21]..

### Self-perception of body weight status

The task for the young people surveyed was to assess whether they are definitely too fat, slightly too fat, just right, too slim, definitely too slim. For further analysis the variable was re-coded into three categories by combining marginal answers.

### Self-perception of body weight status and BMI

The main outcome variable is a derivative of objective and subjective assessment of body weight. Four groups were distinguished with regard to the self-awareness of weight. Among the four, two have correct perception of their body weight and the other two distinct misperception of their body weight:

− group 1: Correct perception of normal weight (BMI normal or below the norm, respondents do not consider themselves too fat);
− group 2: Correct perception of overweight (BMI above norm, respondents consider themselves too fat );
− group 3: Overestimation of body weight (normal BMI, respondents consider themselves too fat)
− group 4: Underestimation of body weight (BMI above norm, respondents do not consider themselves too fat).

The first two groups include adolescents who have a correct assessment of their body weight from the perspective of potential overweight. The third and fourth group include persons who have a distorted assessment.

### Physical activity

The MVPA (Moderate – to - Vigorous Physical Activity) indicator used in HBSC studies since 2002 and based on the Prochaska screening test [22] has been used to assess the level of physical activity. Young people were asked about the number of days the previous week when they had spent a total of at least 60 minutes a day on physical activity. The question was preceded by an introductory explanation of what is meant by physical activity. The MVPA indicator also embraces physical education classes at school. The current recommendation is that children and young people should take up moderate to vigorous physical activity for a minimum of one hour a day [23].

### Statistical methods

A large part of the analyses is based on simple descriptive statistics. The Analysis of Variance (ANOVA) was used to compare average BMI values. The impact of physical activity on the level of misperceptions between the objective and subjective assessment of body weight was studied using multinomial logistic regression. The dependent variable was divided into four groups by the adequacy of body weight assessment, as described above. The results of logistic regression were presented as odds ratios (OR) with a 95% confidence interval (CI). The SPSS v.17 statistical software was used.

## Results

### Prevalence of excess body weight by objective and subjective measures

The first part of the analysis is associated with the answer to the first and second research question.

Table I presents the percentage of 15-year-old boys and girls in terms of objective (BMI) and subjective (self-perceived) assessment of body weight. In the combined international HBSC sample excess body weight measured by the BMI applied to 15.2% of respondents − 12.5% were overweight, 2.7% were obese. In Poland the percentage of young people with excess body weight was lower than the international average and amounted to 12.3% (10.5% overweight and 1.8% obese). The percentage of young people with excess body weight ranges from 7.1% in the Ukraine to 27.2% in Malta. Poland is 11^th^ in the ranking of countries by increasing percentages (data not presented).

**Table. I:** Subjective and objective weight assessment of 15-year-olds by gender (%). Tabela. I. Subiektywna i obiektywna ocena masy ciała uczniów 15-letnich wg płci (%).

	Objective body mass *Obiektywna masa ciała (N=61808)*		Subjective body mass *Subiektywna masa ciała (N=70513)*		
	underweight & normal *niedowaga oraz norma*	overweight & obesity *nadwaga i otyłość*	too thin *zbyt szczupła*	about right *w normie*	too fat *zbyt gruba*
**Total /*Ogółem***					
Poland/*Polska*	87 .7	12 .3	17 .8	36 .3	45 .9
HBSC average/*Średnia HBSC*	84 .8	15 .2	15 .4	51 .9	32 .7
**Boys/*Chłopcy***					
Poland/*Polska*	81.3	18.7	26.2	44.9	28.8
HBSC average/*Średnia HBSC*	80.9	19.1	20.7	57.4	21.9
**Girls/*Dziewczęta***					
Poland/*Polska*	93.5	6.5	10.4	28.7	60.9
HBSC average/*Średnia HBSC*	88.5	11.5	10.4	46.6	43.0

In Poland the percentage of 15-year-old boys with excess body weight is nearly three times higher than that of girls of the same age (18.7% compared with 6.5%). In the case of boys the level of overweight and obesity is similar to the international average (22^nd^ place in the ranking), while in the case of girls it is significantly lower than average (5^th^ place).

According to international statistics, the percentage of young people who consider themselves too fat is 32.7%- that is two times higher than the percentage with actual excess body weight. This percentage ranges between 7.3% in Macedonia to 45.9% in Poland, which means that Polish 15-year-olds are first in the ranking of decreasing indicators. In Poland and Luxembourg the percentage of 15-year-olds who believe they are too fat clearly exceeds the percentage expected on the basis of the actual percentage of overweight and obese persons in this population. Conversely, in Macedonia and Armenia very low percentages are recorded for those with such an opinion in comparison with the real prevalence of excess body weight. In most countries the percentage of girls who consider themselves too fat clearly exceeds the corresponding percentage for boys. On the international scale this is a two-fold difference (43.0% to 21.9%). In Poland this percentage exceeds the international average for both genders, for girls to a greater extent than for boys.

### The discrepancy between the objective and subjective assessment of body weight among 15-year-olds

The next two research questions relate to the discrepancy between the objective and subjective assessment of body weight and comparisons of international BMI indicators in four groups distinguished from the point of view of the presence or absence of such a discrepancy. Inadequate assessment of body weight means that the person considers him or herself too fat despite having normal body weight or fails to notice the existing excess body weight.

In the combined international sample ¾ of the teenagers surveyed correctly assessed whether or not they were overweight (tab. II). Most of the population (64.1%) are persons who are not overweight and are aware of it. Every tenth respondent (10.6%) has excess body weight and is aware of it. In the remaining two groups of teenagers with an incorrect self-assessment in terms of being overweight, 20.9% considered themselves too fat despite having normal body weight and 4.7% failed to see that they were actually overweight. In Poland the percentage of 15-year-old adolescents with an incorrect self-assessment of their weight is much higher than the international average and is 38.9%. The reason for this is the over-representation of the third group – i.e. persons with a normal body weight who consider themselves too fat.

**Table II:** Body mass index of 15-year-old girls without overweight according to subjective assessment of appearance (N=27712). Tabela II. Indeks masy ciała dziewcząt 15-letnich bez nadwagi wg subiektywnej oceny wyglądu (N=27712).

Countries *Kraje*	Subjective body mass *Subiektywna ocena masy ciała*					
	too thin *zbyt szczupła*		about *w normie*	right	too fat *zbyt gruba*	
	mean BMI *średni BMI*	*SD*	mean BMI *średni BMI*	*SD*	mean BMI *średni BMI*	*SD*
Albania/*Albania*	17.62	1.92	19.04	1.96	20.69	2.10
Austria/*Austria*	17.33	1.32	19.11	1.61	20.51	1.91
Armenia/*Armenia*	17.08	1.79	19.24	2.01	21.37	2.04
Belgium fl./*Belgia fl*.	17.55	1.74	18.91	1.89	20.82	1.94
Belgium fr./*Belgia fr*.	17.23	1.52	19.56	1.85	20.69	1.94
Bulgaria/*Bułgaria*	17.36	2.17	18.97	1.91	20.55	2.00
Canada/*Kanada*	18.07	1.65	20.22	1.89	21.19	1.88
Croatia/*Chorwacja*	17.89	1.33	19.89	1.63	21.04	1.80
Czech Republic/*Czechy*	17.60	1.55	19.68	1.74	20.73	1.86
Denmark/*Dania*	17.94	1.53	19.53	1.78	20.50	2.11
Estonia/*Estonia*	17.71	1.41	19.77	1.72	21.11	1.91
Finland/*Finlandia*	17.96	1.46	20.06	1.82	21.27	1.85
France/*Francja*	17.38	1.48	19.28	1.74	20.76	1.85
Germany/*Niemcy*	17.33	1.51	19.48	1.85	20.58	1.93
Greece/*Grecja*	18.07	1.64	20.09	1.77	21.39	1.65
Greenland/*Grenlandia*	19.05	1.74	20.92	1.97	21.79	1.53
Hungary/*Węgry*	17.57	1.38	19.43	1.93	20.94	1.71
Iceland/*Islandia*	17.58	1.40	20.16	1.90	21.27	1.96
Ireland/*Irlandia*	17.56	1.44	19.06	2.22	20.82	2.00
Israel/*Izrael*	17.83	1.80	19.85	2.04	21.25	1.94
Italy/*Włochy*	17.25	1.58	19.38	1.96	20.82	1.89
Latvia/*Łotwa*	17.67	1.72	19.70	1.69	20.88	1.80
Lithuania/*Litwa*	17.44	1.48	19.25	1.72	20.48	1.82
Luxembourg/*Luksemburg*	17.91	2.18	19.27	1.71	20.59	1.86
Malta/*Malta*	18.33	1.89	20.47	2.26	21.33	2.05
Moldova/*Mołdawia*	18.09	1.80	19.37	1.87	20.85	1.81
Netherlands/*Holandia*	17.74	1.77	19.28	1.82	20.73	1.98
Norway/*Norwegia*	18.07	1.99	19.96	1.94	20.80	1.94
Poland/*Polska*	17.54	1.39	19.10	1.62	20.13	2.00
Portugal/*Portugalia*	18.20	1.71	19.98	1.77	21.29	1.68
Romania/*Romunia*	17.53	1.33	19.26	1.77	20.76	1.72
Russia/*Rosja*	17.23	1.48	19.35	1.73	20.61	2.13
Slovakia/*Słowacja*	17.40	1.58	19.17	1.80	20.80	1.67
Slovenia/*Słowenia*	17.73	1.64	19.58	1.70	20.90	1.80
Spain/*Hiszpania*	17.97	1.78	19.46	1.82	21.07	1.81
Sweden/*Szwecja*	17.88	1.66	19.87	1.85	20.95	1.84
Switzerland/*Szwajcaria*	17.24	1.40	19.28	1.62	20.87	1.72
Ukraine/*Ukraina*	17.62	1.76	19.15	1.82	20.84	1.67
TFY Macedonia/*Macedonia*	18.15	1.84	20.03	2.03	21.73	1.63
England/*Anglia*	17.96	2.11	19.26	2.06	20.76	2.00
Scotland/*Szkocja*	17.45	2.26	19.65	2.04	20.64	2.07
Wales/*Walia*	17.04	2.45	19.78	2.17	20.93	2.02
Total/*Ogółem*	17.69	1.68	19.59	1.88	20.85	1.90

Despite the indicated discrepancies, the subjective assessment of body weight shows a strong correlation with the BMI, which clearly increases in all the countries in successive groups of young people who consider themselves: too slim, with a correct body weight, and too fat. It may be suspected that major regional and cultural differences exist and affect the assessment of one’s figure. The average body mass index of girls with a normal body weight who consider themselves too fat ranges from 20.1 kg/sq.m. in Poland to 21.8 kg/sq.m. in Greenland. This means that in Poland relatively slimmer girls begin to perceive themselves as too fat (tab. III).

**Table III:** Comparison of objective and subjective assessment of overweight (%). Tabela. III. Porównanie obiektywnej i subiektywnej oceny występowania nadmiaru masy ciała (%).

	No discrepancies in assessment *Brak rozbieżności ocen*		Discrepancies in assessment *Rozbieżność ocen*	
	correct perception of the norm *prawidłowe postrzeganie normy*	correct perception of overweight *prawidłowe postrzeganie nadwagi*	overestimation of body weight *nieuzasadnione postrzeganie nadwagi*	underestimation of body weight *nieświadomość nadwagi*
	group 1	group 2	group 3	group 4
	*grupa 1*	*grupa 2*	*grupa 3*	*grupa 4*
**Total/*Ogółem***				
Poland/*Polska*	51.7	9.3	35.9	3.0
HBSC average/*Średnia HBSC*	64.1	10.6	20.6	4.7
**Boys/*Chłopcy***				
Poland/*Polska*	64.8	12.4	16.4	6.4
HBSC average/*Średnia HBSC*	72.3	11.3	9.7	7.8
**Girls/*Dziewczęta***				
Poland/*Polska*	39.7	6.5	53.8	-
HBSC average/*Średnia HBSC*	57.2	10.0	31.1	1.6

**Fig. 1:**
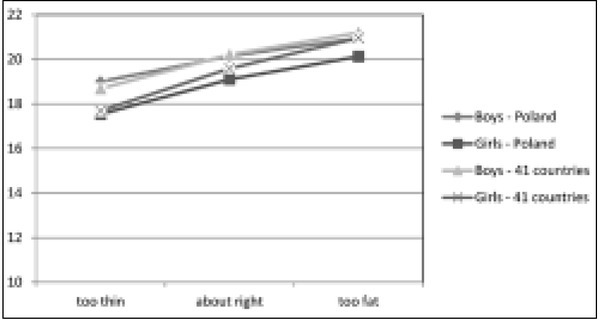
Average body mass index of boys and girls in the non-overweight group according to subjective weight assessment. Ryc. 1. Średni indeks masy ciała chłopców i dziewcząt w grupie bez nadwagi wg subiektywnej oceny masy ciała.

A comparison between Polish data with a combined sample from 41 countries reveals slight differences in the level of BMI in boys with a normal body weight at various levels of subjective assessment of body weight. An important difference was recorded only in the group of boys who considered themselves too slim (p=0.035). Polish boys who consider themselves too slim have a higher BMI than their peers from other countries. In the case of girls with a normal body weight significantly lower BMI values (p<0.001) than in other countries are recorded in the group who consider their body weight to be too high or “just right” (fig. 1).

**Fig.2:**
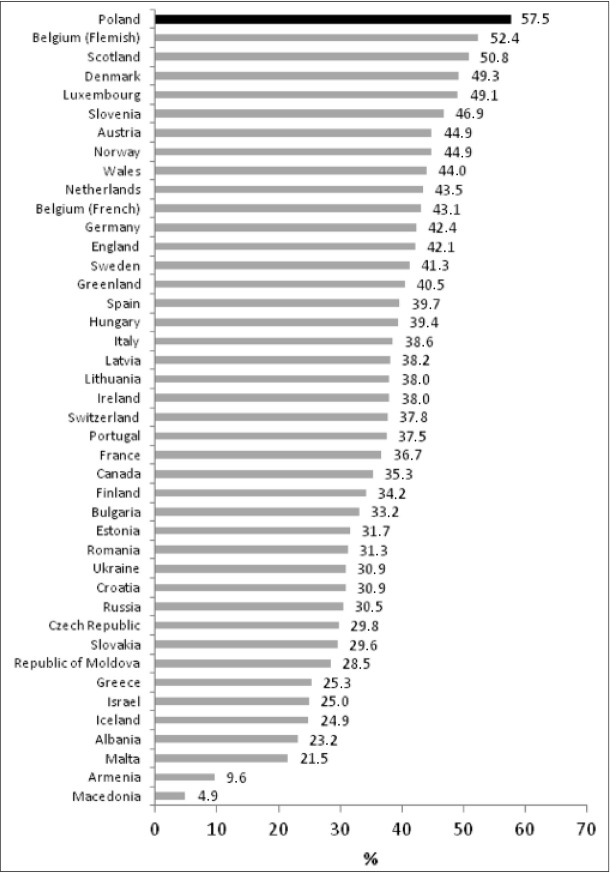
The percentage of 15-year-old girls with BMI in normal range who consider themselves overweight. Ryc. 2. Odsetek dziewcząt uważających się za zbyt grube w grupie bez nadmiaru masy ciała.

**Fig. 3:**
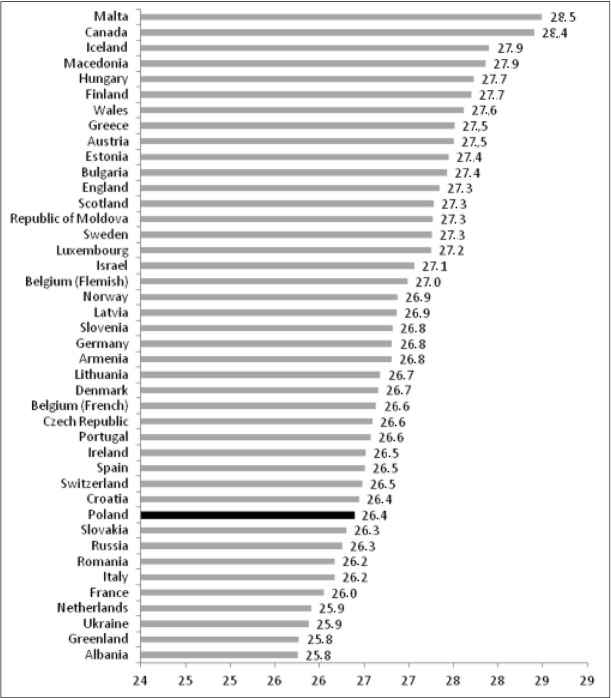
The percentage of 15-yer-old boys with excess weight who consider their body mass as normal. Ryc.3. Odsetek chłopców z nadmiarem masy ciała, sądzących, że ich masa ciała jest prawidłowa.

When analyzing individual data obtained in particular countries, the unfavourable position of Poland in terms of the percentage of 15-year-old girls without excess body weight who consider themselves too fat must be noted. This percentage ranges from 4.9% in Macedonia to 57.5% in Poland (fig. 2). Conversely, the percentage of boys who do not notice their overweight (in the group with actual overweight) shows minor variation between countries, ranging from 25.8% in Albania to 28.5% in Malta. Poland ranks tenth in terms of increasing percentages (fig. 3).

### Physical activity and correct body weight assessment

Recommendations as to vigorous physical activity (MVPA) were fulfilled by 15.8% of those polled in the studied international group. The percentage ranged from 7.7% in Israel to 23.4% in Moldova. Poland, with a 17.8% frequency, occupied a fairly good place, 31^st^ in the international ranking of 42 countries by increasing values of the MVPA indicator (data not presented).

A significant correlation was demonstrated between the level of physical activity, MVPA, and membership in groups with varying correctness of self-assessment of body weight. It has been observed that with improving physical activity there was an increase in the percentage of young people with appropriate body weight who had an adequate assessment of their body weight as being “just right” (group 1). Only in the case of boys was there an increase in the percentage of those who having an objective excess of body weight did not notice it in their self-assessment (group 4). At the same time, along with an improvement of the physical activity indicator, the percentage of adolescents from group 2 decreased (in both genders) (those who acknowledge actually excessive body weight), as did the percentage of adolescents in group 3 (those who unjustifiably considered themselves too fat).

The strength of the correlation between the level of moderate to vigorous physical activity and the correct assessment of body weight was also measured by the odds ratio, on the basis of estimation models of multinomial logistic regression. The reference category was the first group: adolescents without excess body weight who objectively consider it to be correct, and the reference group from the point of view of physical activity was MVPA=7 days. The models were estimated separately for boys (tab. IV) and girls (tab. V). A low level of physical activity was associated with a significantly higher risk of finding oneself in group 2 and 3, higher in the case of group 2. A tendency of declining OR ratios with deteriorating physical activity was found in both genders. It was also demonstrated that the level of physical activity is not associated with a failure to notice excess weight by young people who are objectively confirmed as overweight (group 4).

**Table IV:** Group membership according to the correctness of body mass assessment by physical activity level in 15-year-old boys. Tabela IV. Przynależność do grup wg adekwatności oceny masy ciała wg poziomu aktywności fizycznej w grupie chłopców 15-letnich.

		MVPA			
		day	2-4 days	5-6 days	7days
		***0-1 dzień***	***2-4 dni***	***5-6 dni***	***7 dni***
Group 1/*Grupa 1*	%	65.4	68.0	73.3	77.3
Correct perception of the norm *Prawidłowe postrzeganie normy*	reference group/ *grupa odniesienia*				
Group 2/*Grupa 2*	%	14.8	14.0	9.5	6.3
Correct perception of overweight	OR	2.77	2.52	1.59	1.00
*Prawidłowe postrzeganie nadwagi*	CI	2.40-3.20	2.25-2.83	1.40-1.80	(ref.)
Group 3/*Grupa 3*	%	12.2	11.2	8.9	7.3
Overestimation of body weight	OR	1.99	1.75	1.29	1.00
*Nieuzasadnione postrzeganie nadwagi*	CI	1.72-2.30	1.57-1.96	1.14-1.46	(ref.)
Group 4/*Grupa 4*	%	7.6	6.8	8.3	9.0
Underestimation of body weight	OR	0.99	0.84	0.97	1.00
*Nieświadomość nadwagi*	CI	0.84-1.17	0.77-0.96	0.87-1.10	(ref.)

MVPA – moderate-to-vigorous physical activity/*aktywność fizyczna intensywna do umiarkowanej*. ^*^OR – odds ratio estimated by multinomial logistic regression/*iloraz szans z modelu regresji logistycznej wielomianowej*.

**Table V:** Group membership according to the correctness of body mass assessment by physical activity level in 15-year-old girls. Tabela V. Przynależność do grup wg adekwatności oceny masy ciała wg poziomu aktywności fizycznej w grupie dziewcząt 15-letnich.

		MVPA			
		day	2-4 days	5-6 days	7days
		*0-1 dzień*	*2-4 dni*	*5-6 dni*	*7 dni*
Group 1/ *Grupa 1*	%	54.6	55.5	58.3	63.2
Correct perception of the norm *Prawidłowe postrzeganie normy*	reference group*/ grupa odniesienia*				
Group 2 /*Grupa 2*	%	11.4	10.5	9.0	6.5
Correct perception of overweight	OR	2.01	1.83	1.49	1.00
*Prawidłowe postrzeganie nadwagi*	CI	1.71-2.37	1.58-2.12	1.26-1.76	(ref.)
Group 3/ *Grupa 3*	%	32.4	32.5	30.9	28.6
Overestimation of body weight	OR	1.31	1.30	1.18	1.00
*Nieuzasadnione postrzeganie nadwagi*	CI	1.19-1.44	1.19-1.41	1.07-1.29	(ref.)
Group 4/ *Grupa 4*	%	1.6	1.5	1.8	1.7
Underestimation of body weight	OR	1.12	1.02	1.14	1.00
*Nieświadomość nadwagi*	CI	0.80-1.58	0.76-1.37	0.82-1.57	(ref.)

MVPA – moderate-to-vigorous physical activity/*aktywność fizyczna intensywna do umiarkowanej* ^*^OR – odds ratio estimated by multinomial logistic regression/*iloraz szans z modelu regresji logistycznej wielomianowej*

After introducing the country of residence variable into the model (Poland as opposed to the remaining 42 countries) the risk of being in group 3 (adolescents who overestimate their body weight) increased in Poland [OR=2.20; CI (1.96-2.41)].

## Discussion

The object of the analysis was the assessment of the position of Polish 15-year-olds against the background of their peers from 42 countries of Europe and North America from the point of view of objective and subjective body weight. The authors indicated significant discrepancies between self-perception and objective measurements of body weight. Emphasis in the analyses was placed on the differences dependent on gender. The analyses indicated that the percentage of 15-year-old overweight boys in Poland is nearly three times higher than girls of the same age (18.7% compared with 6.5%). In the case of boys this is a result that is close to the international average (22^nd^ place in the ranking). Among girls the prevalence of overweight and obesity is evidently less frequent than the international average (5^th^ place). A similar difference in their observations of excess body weight depending on the respondent’s gender was recorded by scientists in Japan, who conducted a 6-year study on a group of 16 thousand students. It was demonstrated that Japanese boys are fatter than girls of the same age, the prevalence of overweight was approximately 23% for boys and approximately 18% for girls, and in the case of obesity approximately 7% for boys and 4% for girls [24]. On the basis of a systematic review of the literature embracing scientific tests with a global circulation published since mid-2012 concerning the prevalence of overweight and obesity among children and young people aged 10-19 years, Del Mar Bililioni et al. [25] confirmed that overweight is a frequent phenomenon in this population and to a greater degree affects teenage boys.

On the basis of the results obtained, it is to be concluded that the tendency to overestimate their body weight in their subjective assessment is present among adolescents all over the world and teenage girls are more inclined to do so. Among Polish and adolescents this correlation is even more evident. An analysis of the subjective assessment of body weight indicates that Polish 15-year-old girls rank first on the international list of girls who consider themselves too fat. In the group without excess body weight as many as 57.5% of the girls polled gave this answer. For the sake of comparison, in the Korean study Youth Risk Behavior Web-based Survey, 47.5% of teenage girls and 22.2% of teenage boys consider themselves too fat, while only 11.9% and 4.5%, respectively, are actually overweight or obese [26]. Studies concerning satisfaction with one’s appearance in relation to body weight among teenage boys and girls were also conducted by Kantanista et al. They demonstrated an important correlation between the BMI, gender and satisfaction with one’s body. Among boys satisfaction was greater in the group having normal body weight. Underweight girls expressed greater satisfaction with their appearance than those whose BMI was normal or showed overweight [27].

Another problem which must be noted is that of the failure to acknowledge excess body weight by boys, which appears among 7.8% of male teenagers in Poland (and as many as 26% of overweight boys). This is an aspect of the wrong self-perception of body weight that is essentially neglected in the literature, although it is important from the point of view of preventing obesity. It may be assumed that with regard to boys requirements are different and they do not feel stigmatized due to being slightly overweight [28]. More emphasis is also placed on the perception of overweight from the parents’ perspective and with regard to younger children [29].

If we compare the results obtained concerning the prevalence of overweight and obesity with the self-perception of body weight by adolescents, we observe that those girls who most frequently consider themselves too fat come from countries where the percentage of overweight or obese teenage girls is relatively low. Conversely, boys who fail to see their excess body weight, come from countries where the problem of overweight and obesity is most frequent (Malta, Canada). It may be supposed that the actual body appearance that dominates in those countries influences the shaping of opinions concerning a socially acceptable image, while the social norms determine how teenagers perceive their own body and its size.

Analyzing the incoherence between self-assessment of body weight by young people and objective indicators, we must also consider other reasons for the declared discrepancies. According to longitudinal studies by Dion et al. girls tend to be more dissatisfied with their body than boys. The level of satisfaction with their appearance may be projected on the basis of earlier measurements. A large number of girls whose body weight is normal or below norm wish to be slimmer, while some boys would like to be fatter [30]. This phenomenon is associated with internalized standards of a slim body for girls and a muscular body for boys propagated in the mass media [31] and the Internet, especially in the social media widely used by teenagers nowadays [32]. It is to be noted that girls are more prone to media influence, especially as the norms and messages are more frequently addressed to girls. Other risk factors for the development of problems with self-perception of the body by girls are associated among others with comparing oneself with others, low self-esteem, the peer group (teasing about appearance, modeling of peer behaviour, talking about appearance), the family (parents’ comments, modeling of parental behaviour, especially the mother). Among factors associated with experiencing violence, BMI values and genetic determinants are also to be noted. Moreover, in the case of boys depression and social roles associated with gender [33] are mentioned. When looking for the reasons why in some countries girls with a normal body weight are more likely to consider themselves too fat and boys with a higher body weight fail to see the excess, the standards of body appearance prevalent in various countries and cultures must definitely be taken into account [34].

It is dificult to unequivocally determine the main reason for the disadvantageous situation of Polish girls in the international ranking. Based on theoretical models which describe the determinants of body self-perception by teenagers it would be necessary to examine the connection between potential factors affecting the negative self-assessment of appearance and their mutual interactions, and to analyze them in the international context.

The value added of the study is the analysis of the discrepancy between objective and subjective body weight in the context of the level of physical activity, which corresponds with the work of other authors. Golan M. et al. demonstrated that physical activity has an impact on the correct assessment of body weight by teenagers. However, this impact was most significant in the group of young people with appropriate body weight [35]. Sampasa-Kanyinga et al. came to similar conclusions in their study. The young people surveyed who fulfilled the physical activity recommendations were less frequently dissatisfied with their body weight than those who did not fulfill such recommendations (28.8% vs 37.5%). The first group was also more likely to perceive themselves as persons with appropriate body weight (71.2% vs 62.5%) [36].

On the basis of our investigations we may also conclude that physically active young people (with normal body weight) are less likely to consider themselves too fat, which can be linked with higher self-esteem among physically active adolescents in general [37].

The correlation between physical exercise and the perception of one’s body by young people was also studied by Czech scientists, although they adopted physical activity as an outcome variable. Their research confirmed the existence of such a correlation. They demonstrated, for example, that dissatisfaction with their own body among girls did not affect physical activity, while among boys it was associated with an insufficient dose of daily exercise. Conversely, studies of Spanish adolescents indicated that dissatisfaction with the appearance of one’s body negatively correlates with the level of physical activity, irrespective of gender [38].

Young people who perceive themselves as normal or slim more frequently follow the physical activity recommendations. It is likely that as a result of physical activity young people without excess body weight have a smaller tendency to put on weight. Conversely, excessively fat teenagers have a problem with being active, which contributes to maintaining excess weight. It may also be concluded that intervention programs aiming to promote physical activity and sport among young people ought to take into account the subjective assessment of their appearance and the factors which determine it. Only then will such programs have a chance of success.

## Main findings

significant discrepancies between the objective and subjective assessment of body weight by adolescents exist in all countries. International data indicate that about 15% of 15-year-olds have excess body weight in terms of the BMI, while twice as many persons of this age consider themselves too fat. In Poland this discrepancy is even more visible.

Polish 15-year-old girls have a major problem with the correct assessment of their body weight. They consider themselves too fat much more often than their peers from 42 other countries, although this is not confirmed in BMI categories. In the case of boys the situation is similar; only their peers from the Netherlands and Luxembourg had a less correct self-perception of body weight regarding overestimation.

However, the self-perception of body weight by adolescents strongly correlates with the BMI. It is to be noted that the average BMI value in homogeneous groups distinguished by self-perception of body weight shows great variation between countries. In Poland, boys who consider themselves too slim have higher BMI values than in many other countries. The reverse situation is observed among girls; significantly lower BMI values are recorded in the group which considers their body mass too high or ”just right”. This means that relatively slimmer Polish girls begin to consider themselves too fat.

Another point to be noted is the relatively large group on the international scale of boys who fail to notice their overweight. Their share in the population increases in countries where the phenomenon of overweight or obesity is more frequent. In the population of girls failure to notice actual overweight occurs very infrequently among 15-year-old girls, in Poland it is practically non-existent.

The level of physical activity is a factor which modifies the discrepancy between objective and subjective assessment of body weight by teenagers. Among less physically active young people the risk of belonging to the overweight group (those with a realistic assessment of their body weight) and to the group with normal body weight (wrongly identifying themselves as overweight) increases. In both cases the effect of physical activity is more strongly visible among boys.
